# The interactive association of smoking and drinking levels with presence of periodontitis in South Korean adults

**DOI:** 10.1186/s12903-016-0268-y

**Published:** 2016-08-25

**Authors:** Mira Lee, Yoon-Hyeong Choi, Jun Sagong, Sol Yu, Yongbae Kim, Dongjae Lee, Sungroul Kim

**Affiliations:** 1 Department of Environmental Health Sciences, Soonchunhyang University, Soonchunhyang-Ro 22, Asan, Chungnam 336-745 South Korea; 2 Graduate School of Medicine, Gachon University, Incheon, South Korea; 3 Preventive Medicine and Public Health, College of Medicine, Yeungnam University, Daegu, South Korea; 4 Department of Preventive Medicine, Soonchunhyang University, Cheonan, South Korea

**Keywords:** Smoking, Drinking, Interaction, Periodontitis

## Abstract

**Background:**

Periodontitis is a chronic and long-lasting low-grade inflammatory disease. Numerous studies have shown that the severity of periodontitis rose when there was an increase in the amount of smoking or alcohol consumption. However, as periodontitis known as a chronic disease, it is important to consider not only the amount but “duration” with frequency i.e., rates, of smoking or drinking. This study assessed impacts of the amount and duration of smoking and drinking on periodontal health in Korean adults. We also investigated whether or not there is an interactive effect of smoking and drinking on periodontal health.

**Methods:**

Under a cross-sectional study design, we used data from the fourth and fifth the Korean National Health and Nutrition Examination Survey (KNHANES) sessions (2008–2010). A total of 18,488 subjects (over 19 years) answered both smoking and drinking status and were given the periodontal examination. Periodontal health status was determined by the community periodontal index (CPI) developed by the World Health Organization (WHO). According to the WHO guidelines, if a participant’s CPI was 3 or larger, we classified the person as a case of periodontitis. Participants with a CPI < 3 were assigned to the control group.

**Results:**

Prevalence of periodontitis for self-reported smokers or drinkers in South Korea was 35.0 or 28.0 %, respectively. We observed 1.20 (0.93~1.56) of odds ratio (95 % CI) for prevalence (POR) of periodontitis for those smoked <13 pack-year (PY) and drank ≥6.8 glass-year (GY). And we had POR of 1.91 (1.34~2.73) for those smoked ≥13 PY and drank <6.8 GY, compared to those nonsmoking nondrinkers. The observed POR of 2.41 (95 % CI: 1.94–3.00), for those smoked ≥13 PY and drank ≥6.8 GY, was higher than a multiplicative effect estimated, i.e., 1.20 (0.93~1.56) [those smoked <13 PY and drank ≥6.8 GY] × 1.91 (1.34~2.73) [those smoked ≥13 PY and drank <6.8 GY], or 2.29.

**Conclusions:**

We observed a multiplicative interactive effect of smoking and drinking on periodontal status among Korean adults.

## Background

Periodontitis is a chronic and long-lasting low-grade inflammatory disease [1] and the prevalence of periodontitis is particularly high in the adult population. According to the Korean health statistics, periodontitis was one of the most common reasons for visiting dental clinics in 2011 [2].

The risk factors of periodontitis include smoking, alcohol consumption, obesity, and diabetes mellitus [3]. Many studies have shown the relationship between periodontal condition and smoking status [4–8]. In addition, several studies noted that alcohol consumption has been shown to increase severity of periodontitis, even when other lifestyle factors, including smoking, have been adjusted for [9–13].

Recently, numerous cross-sectional studies and a prospective study have shown that the severity of periodontitis rose when there was an increase in the amount of smoking or alcohol consumption [14–17]. However, as periodontitis known as a chronic disease, it is important to consider not only the amount but “duration” with frequency i.e., rates, of smoking or drinking. Previous studies reported a positive association between change in Community Periodontal Index (CPI) scores with duration of smoking or drinking as well as their amount [6, 7, 18]. In addition, several investigators have showed that there was an interaction smoking and drinking for periodontitis [9, 14]. However, Most of these previous studies were conducted in Western countries where smoking or drinking behaviors maybe different from in South Korea. They didn’t consider duration of smoking and drinking when they evaluated whether there was an interaction smoking and drinking consumption for periodontitis.

Therefore, this study assessed impacts of the amount and duration of smoking and drinking on periodontal health in Korean adults using a data from KNHANES 2008 to 2010. We also investigated whether or not there is an interactive effect of smoking and drinking on periodontal health.

## Methods

### Data source

This study was conducted with a cross-sectional study design using the data corresponding to the fourth and fifth KNHANES sessions. The KNHANES IV and V data (2008–2010) were comprised of nationally representative samples and were extracted from standard household surveys using a systematic sampling method that was adjusted to the number of households while accounting for region, administration district, and type of residence (apartment or individual house) in South Korea. The sampling protocol for KNHANES was designed to involve a complex, stratified, multistage, probability-cluster survey of a representative sample of the non-institutionalized civilian population in South Korea. Trained interviewers visited the subjects in their homes and administered a standardized health examination and questionnaire. More details on the sampling methodology and the data of KNHANES IV and V are available from the Guidelines for Use of KNHANES IV and V Sampling Frames [19].

### Study population

**Fig. 1:**
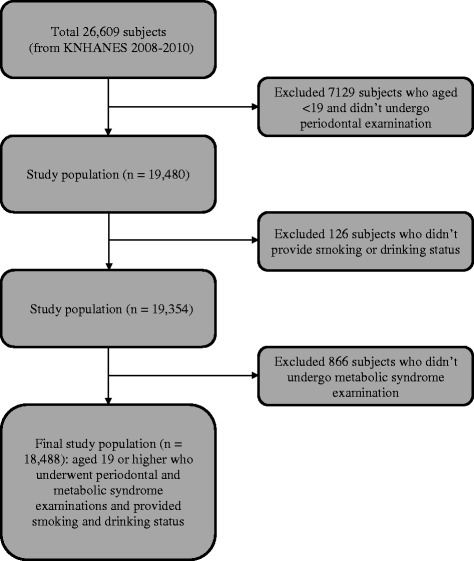
Flow chart for selecting process of study population

A total of 26,609 subjects participated in KNHANES from 2008 to 2010, and 19,480 subjects aged over 19 were examined for the periodontal examination. Seven thousand one hundred twenty nine subjects aged less than 19 were excluded. Participants who did not provide the smoking and drinking questionnaire (*n* = 126) nor undergo a health examination (*n* = 866) were excluded. The final sample for the present study comprised 18,488 subjects (Fig. 1).

We defined a smoker as a person who had smoked more than five packs of cigarettes (100 cigarettes) in his/her life and currently smoked more than 1 cigarette/day at the time of the interview [20]. Everyone else was classified as a non-smoker. Smokers were asked the number of cigarettes that they smoked per day and how many years they had smoked. In our study “cigarettes per day (CPD)” and “pack years (PY)” were used to measure of the short- or long- term smoking rate, separately. PY for current smokers was calculated by multiplying the life-time smoking period (year = 365 days) by the number of packs per day.

A drinker was defined as a person who self-reported as a drinker and who had drunken at least one glass of alcohol per month over the past year. Everyone else was classified as a nondrinker. One glass is equivalent to a glass of alcohol without distinguishing between beers, so-ju or foreign liquors as KNHANES did not distinguished them. Drinkers were asked the number of glasses consumed per month as well as lifetime drinking period. The number of glasses per day (GPD) was calculated by dividing the total number of glasses drunk per month by 28 days (4 weeks multiplied by 7 days per week) as an indicator of short-term drinking levels. Glass years (GY) was calculated by multiplying glasses per day by the life-time drinking period (year = 365 days). In our study, GY was used an indicator of long-term drinking levels.

### Assessment of periodontitis

KNHANES used the community periodontal index developed by the World Health Organization (WHO) to assess periodontal condition [21]. According to WHO guidelines, if a participant’s CPI was ≥3 the person is counted as a case of periodontitis, indicating that more than one site had a 3.5 mm pocket or larger in the index teeth. Participants with a CPI < 3 were assigned to the control group.

### Statistical analysis

This study was conducted with a cross-sectional study design. A pooled survey weight for the data sets from 2008, 2009, and 2010 was applied to increase statistical power and account for the complex sampling design [22]. Data were analyzed using SPSS survey procedures. A chi square test was used to show prevalence for periodontitis depending on the distribution of demographic characteristics, oral health behaviors, oral health status, smoking, and drinking. To assess whether there was an association between short-term and long-term consumption rate of smoking or drinking and the prevalence of periodontitis, logistic regression analyses were conducted [23]. After stratification of our data by smoking and drinking levels, we examined whether or not there is an interactive effect of the level of smoking on the degree of the presence of periodontitis depending on the level of drinking [24].

Three different models were run in logistic regression analyses. The first model was developed to assess the association of the existence of periodontitis with smoking or drinking status using binary variables (e.g., smokers vs. nonsmokers; drinkers vs. nondrinkers). The second model evaluated the association of existence of periodontitis with categorical variables (CDP, PY, GPD and GY levels) separated with respect to median values distinguishing light short- or long-term smokers (CDP: <15, PY:<13) or drinkers (GPD: <0.5, GY: <6.8) from heavy short- or long-term smokers (CDP: ≥15, PY:≥13) or drinkers (GPD: ≥0.5, GY: ≥6.8). In the final model, we examined whether or not the association of prevalence of periodontitis with short- or long-term consumption rates of smoking, obtained from the second model, can be changed interactively by those of drinking, or vice versa. Nonsmokers and nondrinkers were the reference group for the final model. In the final model, we included subjects who smoked and drank at the same time only to evaluate the interactive effect.

In this study, we controlled demographic characteristics, oral health behaviors, and oral health status as covariates. Demographic characteristics included sex, age, education level, monthly household income level, and marital status. Characteristics of oral health behaviors and oral health status included recent dental visit, use of oral hygiene devices, frequency of daily tooth brushing, and the number of decayed, missing, or filled permanent teeth (DMFT).

Our study design and approaches were evaluated (201407-BM-019-01) and our research approaches were exempt from IRB review by the Ethics Committee of Soonchunhyang University, South Korea.

## Results

Table 1 shows the distribution of prevalence of periodontitis according to demographic and socioeconomic characteristics and oral health status. The prevalence of periodontitis for male was 34.7 % while that for female was 23.6 %. The prevalence was 4 times higher among those aged over 60 years (48.8 %) than that for the group whose aged 19–39 years (12.0 %).

**Table 1:** Prevalence for periodontitis depending on the distribution of demographic characteristics, oral health behaviors, oral health status, smoking level, or drinking levels

Characteristic		Periodontitis prevalence (%)	*p*-value
Sex	Male	34.7	<0.001
	Female	23.6	
Age	19–39 years	12.0	<0.001
	40–59 years	39.4	
	≥60 years	48.8	
Education level	≤ Middle school	46.1	<0.001
	≥ High school	22.7	
Household income	1st and 2nd quartile	33.7	<0.001
	3rd and 4th quartile	26.1	
Marital status	Married	34.2	<0.001
	Single	9.1	
	Widowed or divorced	40.2	
Recent dental visit	Yes	27.5	<0.001
	No	32.5	
Use of oral hygiene device	Yes	24.2	<0.001
	No	34.2	
	< 10	29.3	0.623
	≥ 10	28.8	
Daily frequency of tooth brushing	< 3	33.0	<0.001
	≥ 3	24.4	
The number of present teeth (including third molar)	< 28	41.9	<0.001
	≥ 28	21.0	
Smoking	Yes	35.0	<0.001
	No	27.0	
	0 CPD	27.0	<0.001
	<15 CPD	25.7	
	≥15 CPD	41.9	
	0 PY	27.0	<0.001
	< 13 PY	18.2	
	≥ 13 PY	51.7	
Drinking	Yes	28.0	<0.001
	No	33.8	
	0 GPD	33.4	<0.001
	< 0.5GPD	24.9	
	≥ 0.5 GPD	31.0	
	0 GY	33.1	<0.001
	< 6.8 GY	21.0	
	≥ 6.8 GY	35.1	

*CPD* “cigarettes per day”, short-term smoking rate, *PY* “pack years”, long-term smoking rate

*GPD* “glasses per day”, short-term drinking rate, *GY* “glass years”, long-term drinking rate

^a^Sum of percentage values may not be 100 % due to missing information

The prevalence was 1.2 times higher among participants who had not visited a dental clinic in the past year (32.5 %), compared to other participants (27.5 %). Groups of participants who did not use oral hygiene devices or whose frequency of tooth-brushing was less than three times per day had prevalence of periodontitis of 34.2 or 33.0 %, respectively, which were approximately 1.4 times higher than the prevalence in groups of participants who used oral hygiene devices or whose frequency of tooth brushing was greater than three times per day (24.2 or 24.4 %). The prevalence (41.9 %) among persons with below 28 teeth was approximately 2 times higher than that (21.0 %) among persons with 28 or more teeth. The prevalence of periodontitis for self-reported smokers and drinkers was 35.0 and 28.0 %, respectively.

The odds ratio of prevalence (POR) and 95 % confidence interval (95 % CI) for periodontitis, according to the short- or long-term consumption rate of smoking and drinking using median values for light and heavy cut-points, is shown in Table 2. Risk of presence of periodontitis for subjects who smoked ≥15 CPD or ≥13 PY, compared to nonsmoking nondrinkers, were 2.12 (1.63–2.77) or 1.96 (1.46–2.63) times higher while the PORs for subjects who smoked less than 15 CPD or 13 PY were 1.42 (1.07–1.88) or 0.99 (0.72–1.36). PORs of periodontitis by smoking levels showed dose response relationships. For those who drank <0.5 and ≥0.5CPD, or <6.8 and ≥6.8GY, PORs were 1.00 (1.00–1.00) and 0.99 (0.80–1.23) or 1.00 (1.00–1.00) and 1.22 (1.96–1.56) times higher, compared to nonsmoking nondrinkers.

**Table 2:** Odds ratios for presence of periodontitis according to the level of smoking or drinking in the short- and long-term

Term	Status	*N*	Classification	Crude	Adjusted^a^
				OR (95 % CI)	OR (95 % CI)
Short term	Ref^b^	5,019,783		1.00	1.00
	Smoking	2,687,615	< 15 CPD	0.73 (0.63~0.84)	1.42 (1.07~1.88)
		3,511,323	≥ 15 CPD	1.52 (1.35~1.71)	2.12 (1.63~2.77)
	Drinking	1,544,110	< 0.5 GPD	0.70 (0.63~0.78)	1.00 (1.00~1.00)
		4,654,828	≥ 0.5 GPD	0.94 (0.86~1.04)	0.99 (0.80~1.23)
Long term	Ref^b^	5,001,884		1.00	1.00
	Smoking	3,194,279	< 13 PY	0.47 (0.40~0.54)	0.99 (0.72~1.36)
		2,965,034	≥ 13 PY	2.25 (1.99~2.55)	1.96 (1.46~2.63)
	Drinking	1,656,042	< 6.8 GY	0.56 (0.50~0.63)	1.00 (1.00~1.00)
		4,503,271	≥ 6.8 GY	1.14 (1.03~1.25)	1.22 (0.96~1.56)

^a^Adjusted for sex, age, education level, monthly household income, marital status, recent dental visit, use of oral hygiene device, the number of decayed, missing, or filled permanent teeth (DMFT), frequency of daily tooth brushing

^b^Nondrinking nonsmokers

**Fig. 2:**
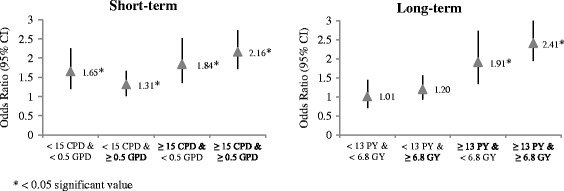
The interactive effect of short-term (*left*) or long-term (*right*) smoking and drinking levels on presence of periodontitis (ORs were obtained after adjusting for variations of demographic characteristics, SES, oral health behaviors, and oral health status)

Table 3 and Fig. 2 show the interactive effect of smoking and drinking consumption on the existence of periodontitis. For those who had similar short-term drinking rate levels (either <0.5 GPD or ≥0.5 GPD, the POR for those heavier smokers (≥15 CPD) showed higher POR levels (1.84 (1.35–2.51) vs. 1.65 (1.20–2.26) for <0.5 GPD group; 2.16 (1.72–2.72) vs. 1.31 (1.02–1.67) for ≥0.5 GPD group), compared to that for those who smoked less than 15 CPD respectively. No matter what their long-term drinking levels (either ≥6.8 GY or <6.8 GY), long-term heavy smokers (≥13 PY) exhibited higher PORs levels (2.41 (1.94–3.00) for ≥6.8 GY or 1.91 (1.34–2.73) for <6.8 GY, respectively), compared to those of long-term light smokers (<13PY) (1.20 (0.93–1.56) for ≥6.8 GY or 1.01 (0.71–1.44) for ≥6.8 GY), respectively, controlling for demographic characteristics, SES and dental hygiene status.

**Table 3:** The interactive effect of short- or long term smoking and drinking levels on presence of periodontitis

Variables	Adjusted	Adjusted
	OR (95 % CI)	OR (95 % CI)
Short-term degree of smoking and drinking (Ref: nonsmoking and nondrinking)		
< 15CPD & < 0.5GPD	1.65 (1.20~2.26)	
< 15CPD & ≥ 0.5GPD	1.31 (1.02~1.67)	
≥ 15CPD & < 0.5GPD	1.84 (1.35~2.51)	
≥ 15CPD & ≥ 0.5GPD	2.16 (1.72~2.72)	
Long-term degree of smoking and drinking (Ref: nonsmoking and nondrinking)		
< 13PY & < 6.8GY		1.01 (0.71~1.44)
< 13PY & ≥ 6.8GY		1.20 (0.93~1.56)
≥ 13PY & < 6.8GY		1.91 (1.34~2.73)
≥ 13PY & ≥ 6.8GY		2.41 (1.94~3.00)
Age^a^	1.05 (1.05~1.06)	1.05 (1.04~1.06)
Sex (Ref: Female)		
Male	1.68 (1.39~2.03)	1.59 (1.321.91)
Education level (Ref: ≥ High school)		
≤ Middle school	1.18 (0.98~1.43)	1.18 (0.98~1.43)
Household income (Ref: 3rd, 4th quartile)		
1st and 2nd quartile	1.03 (0.89~1.19)	1.03 (0.90~1.19)
Marital status (Ref: Married)		
Single	0.44 (0.34~0.57)	0.51 (0.39~0.68)
Widowed or divorced	0.83 (0.67~1.04)	0.86 (0.69~1.08)
Recent dental visit (Ref: Yes)		
No	1.03 (0.87~1.21)	1.03 (0.87~1.21)
Use of oral hygiene device (Ref: Yes)		
No	0.98 (0.84~1.15)	1.00 (0.86~1.17)
Number of DMFT^a^	0.98 (0.97~1.00)	0.98 (0.97~1.00)
Frequency of daily tooth brushing^a^	1.01 (0.94~1.08)	1.01 (0.94~1.08)

*DMFT* decayed, missing, or filled permanent teeth

*CPD* “cigarettes per day”, short-term smoking rate, *PY* “pack years”, long-term smoking rate

*GPD* “glasses per day”, short-term drinking rate, *GY* “glass years”, long-term drinking rate

^a^continuous variable

Strong interactive effects were obtained when analyzing long-term smoking and drinking rate variables, i.e., calculated by GPD or GY (Fig. 2). The observed POR of 2.41 (1.94–3.00), estimated with long term indicators, for those smoked ≥13 PY and drank ≥6.8 GY, was higher than a multiplicative interactive effect estimated, i.e., 1.20 (0.93–1.56) × 1.91 (1.34–2.73), or 2.29 while we could not obtain such effect with short-term indicators (Fig. 2).

## Discussion

This study found that there was a positive association of study participants’ short-and long-term smoking rate with prevalence of periodontitis. Additionally, we observed an interactive effect of smoking on the prevalence of periodontitis among Korean adults.

### The effect of smoking on the presence of periodontitis

The study conducted in Japan similarly revealed that the PORs (95 % CI) of PD were 1.51 (0.95–2.22), 1.58 (1.13–2.22) and 2.81 (1.96–4.03) among smokers consuming 1–19, 20 or 21 or more CPD, respectively, adjusted for age and alcohol [8]. Another Japanese study also confirmed such dose–response association of smoking with periodontitis; the adjusted ORs of subjects who smoked 0.1–9.9, 10.0–19.9, 20.0–29, ≥30 PY on periodontitis were 1.15 (0.38–3.52), 2.00 (0.86–4.66), 2.27 (0.92–5.63), 3.96 (1.59–9.82) respectively [10]. Even after variables of alcohol consumption were taken into consideration, the ORs of subjects for periodontitis were further increased. A study conducted in Korea also identified that the adjusted ORs (95 % CI) of subjects who smoked <285, 285–660, >660 PY on presence of periodontitis, compared to nonsmokers, were 0.94 (0.53–1.66), 1.39 (0.88–2.19), 1.78 (1.18–2.68) [5].

Several studies showed that duration for smoking consumption increased prevalence of periodontitis; One study reported that the number of sites with pockets measuring 4 mm or more and loss of attachment of 4–5 mm were found to be significantly higher in smokers, especially with increasing frequency and duration of smoking, in comparison to nonsmokers, or in whom it was considerably lower [18]. Another study also reported that tobacco affected most periodontal clinical variables studied, depending on the number of years of consumption [7].

This study examined the quantity of smoking measured in CPD or PY as a short- or a long-term smoking indicator. There have been limited studies examining the effects of smoking quantity on periodontitis, especially using PY as an indicator of long-term smoking. Therefore, our study may be important in that it takes a novel approach that accounts for factors of long term duration and quantity.

### The effect of drinking on the presence of periodontitis

Our study did not find a significantly positive association between drinking with PORs among Korean. Previous studies in other countries have reported inconsistent results. One study conducted in Brazil found that the range of ORs of alcohol consumption for presence of periodontitis among non-smokers were from 1.22 (1.02–1.63) to 3.02 (1.51–5.96) [14]. A study in United States also found the ORs range of clinical attachment loss rose along with increased alcohol consumption per week [16]. However, a study conducted in Japan showed that drinking in males (non-drinker, 1–20, >20 g/day) did not affect on the presence of periodontitis [8]. The study conducted in Japan explained such differences in the impact of alcohol on periodontitis could be related to ethnicity due to polymorphism of aldehyde dehydrogenase2 (ALDH2) genotypes [10, 15]. Above two Japanese studies may support our results, which showed no impact of short- or long-term alcohol consumption on periodontitis for Koreans, whose genotype may be more similar to Japanese rather than Brazilians or Americans. This potential relationship could be explained by alcohol’s adverse effect on host defense (defective neutrophil function and complement deficiency), clotting mechanism (defective prothrombin and vitamin K activity), bone metabolism (increased desorption, decreased formation), healing (vitamin B-complex and protein deficiency) and a direct toxic effect on periodontal tissues [16, 25, 26]. However, to evaluate the association of polymorphism of ALDH2 genotypes with presence of periodontitis after adjustment for drinking status, a future study is necessary.

### The interactive effects of smoking and drinking on the presence of periodontitis

We tested the hypothesis whether there is an interactive effect by the consumption of alcohol and smoking on the prevalence of periodontitis. We found that a high consumption level of smoking contributes to an increase in the presence of periodontitis, regardless of drinking levels (short-term:2.16 (1.72–2.72) vs. 1.31 (1.02–1.67), long-term: 2.41 (1.94–3.06) vs. 1.20 (0.93–1.56)) (Fig. 2).

Especially, with regard to long-term consumption rate of smoking or drinking, we found a significant interaction effect of two risk factors on the presence of periodontitis (*p* < 0.05). The baseline risk of having periodontitis calculated by POR was 1.01 (0.71–1.44) among those who smoked <13PY and drank <6.8GY. As seen in Fig. 2, we observed 1.20 (0.93–1.56) of POR for those smoked <13PY and drank ≥6.8GY and 1.91 (1.34–2.73) for POR for those smoked ≥13 PY and drank <6.8 GY. Theoretically, we could expect a multiplicative interaction with the POR provided, i.e., 1.20 × 1.91, or 2.29. However, the observed POR of 2.41 is higher than a multiplicative effect estimated.

Previous studies conducted in Brazil and in U.S.A. reported the combined effect of smoking and drinking [9, 14]. However, results from these studies were somewhat different from those of our study: the Brazilian study identified that OR of alcohol for presence of periodontitis was approximately two times higher in smokers, compared to nonsmokers [14]. However, the American study reported that the association of alcohol consumption with the presence of periodontitis was not changed by smoking level [9]. As we mentioned above, difference of genotype between Asian and Brazilians or Americans, may explain our results.

### Limitations of this study

Our study has several limitations. First, we could not consider different types of alcoholic beverages (beer, so-ju or foreign liquors) in calculation of alcohol consumption rate because KNHANES did not provide such information. Additionally, an assessment of exposure on smoking and drinking was based on the subject’s questionnaire responses. Questionnaires may result in misclassification due to recall bias. Furthermore, CPI used to evaluate periodontal tissue was likely to be overestimated or underestimated since it was a partial mouth examination. Because misclassification by overestimation or underestimation would be non-differential and CPI method has been validated by WHO and widely used in previous studies, we believe CPI is an appropriate marker to estimate the presence of periodontitis in the present study [5, 27]. Third, the data of KNHANES is cross-sectional study and has a limitation in that we cannot use temporal causality to validate our hypothesis. And the CPI value reports periodontal pocketing status but not attachment loss and is therefore unable to determine the periodontal disease status of the patient. The definition of drinking rate was obtained from self-reporting without distinguishing kinds (beer, whiskey, or etc.) of alcohol.

Therefore, one needs to be careful when interpreting our findings. Careful interpretation may also be necessary when our study result is applied to other ethnic groups rather than Korean or Asian adults.

## Conclusions

From a nationally representative sample of South Koreans, as this study found that prevalence of periodontitis might be affected multiplicatively by interaction of heavy smoking and drinking behaviors, we suggest that the prevention programs of periodontitis carefully consider monitoring of long term smoking and drinking behaviors as well as short-term behaviors when making recommendations to improve oral health in South Korea.

## Abbreviations

CPD: The number of cigarettes per day
DMFT: Decayed, missing, or filled permanent teeth
GPD: The number of glasses per day
GY: Glass years
PY: Pack years
